# Genome wide transcriptional profiling of *Herbaspirillum seropedicae* SmR1 grown in the presence of naringenin

**DOI:** 10.3389/fmicb.2015.00491

**Published:** 2015-05-21

**Authors:** Michelle Z. Tadra-Sfeir, Helisson Faoro, Doumit Camilios-Neto, Liziane Brusamarello-Santos, Eduardo Balsanelli, Vinicius Weiss, Valter A. Baura, Roseli Wassem, Leonardo M. Cruz, Fábio De Oliveira Pedrosa, Emanuel M. Souza, Rose A. Monteiro

**Affiliations:** ^1^Nitrogen Fixation group, Department of Biochemistry and Molecular Biology, Universidade Federal do ParanáCuritiba, Brazil; ^2^Instituto Carlos Chagas, Fundação Oswaldo Cruz, Fiocruz-PRCuritiba, Brazil; ^3^Department of Biochemistry and Biotechnology, Universidade Estadual de LondrinaLondrina, Brazil; ^4^Department of Genetics, Universidade Federal do ParanáCuritiba, Brazil

**Keywords:** *H. seropedicae*, naringenin, RNAseq, plant-bacteria interaction, transcription regulation

## Abstract

*Herbaspirillum seropedicae* is a diazotrophic bacterium which associates endophytically with economically important *gramineae*. Flavonoids such as naringenin have been shown to have an effect on the interaction between *H. seropedicae* and its host plants. We used a high-throughput sequencing based method (RNA-Seq) to access the influence of naringenin on the whole transcriptome profile of *H. seropedicae.* Three hundred and four genes were downregulated and seventy seven were upregulated by naringenin. Data analysis revealed that genes related to bacterial flagella biosynthesis, chemotaxis and biosynthesis of peptidoglycan were repressed by naringenin. Moreover, genes involved in aromatic metabolism and multidrug transport efllux were actived.

## Introduction

Beneficial plant–bacterial interactions promote plant growth and development. During this process molecular changes occur in both partners, and signal molecules are involved in partner communication. In legume-*Rhizobium* interactions, flavonoids released by plant roots induce sets of genes involved in nodulation (Broughton et al., 2000). In addition, flavonoids seem to play a role in other plant bacterial associations. Naringenin stimulates lateral root crack (LRC) colonization of *Arabidopsis thaliana* by *Azorhizobium caulinodans* and *H. seropedicae* a process independent of the *nod* genes (Gough et al., 1997). This flavonoid, secreted by some plants, is a signal molecule that regulates gene expression in bacteria such as *H. seropedicae* (Tadra-Sfeir et al., 2011) and *A. caulinodans* (Webster et al., 1998).

The diazotroph *H. seropedicae* is frequently found in endophytic association with maize (*Zea mays*), rice (*Oryza sativa*), sorghum (*Sorghum bicolor*), sugar cane (*Saccharum officinarum*) and other plants. Inoculation of rice with *H. seropedicae* strains resulted in plant growth promotion and increase in productivity (Baldani et al., 2000; Gyaneshwar et al., 2002). This effect may be due in part to transfer of fixed nitrogen, since ^15^N dilution assays indicate significant N transfer to the host plant (Baldani et al., 2000), and production of phytohormones by the bacteria (Bastián et al., 1998). However, the bacterial genes necessary for the establishment of endophytic interaction and the molecular cues that direct their expression are largely unkown. Previously, we isolated 16 *H. seropedicae* mutant strains in genes regulated by the plant-derived flavonoid naringenin*;* 12 of these were downregulated and 4 upregulated. Four of these genes are involved in the synthesis of the outer membrane of the cell wall, suggesting that changes in the cell surface probably occur during the interaction between *H. seropedicae* and its host plants (Tadra-Sfeir et al., 2011).

To explore which other genes are regulated by naringenin, we determined the transcriptional profile of *H. seropedicae* grown in NFBHP malate medium in the presence or absence of naringenin using RNA-seq.

## Materials and methods

### Bacterial growth

*H. seropedicae* SmR1, a streptomycin resistant strain, was grown at 30°C and 120 rpm in NFbHPN medium (Klassen et al., 1997) in the presence (+Nar) or absence (–Nar) of 100 μM of naringenin containing streptomycin (80 μg.mL^−1^)for 6 h (optical density at 600 nm of 0.8).

### Transcriptome profiling experiments design and analyses

The total RNA was isolated using RiboPure™-Bacteria Kit (Ambion) and treated with DNase I (Ambion) for removal of the remaining genomic DNA. Seven micrograms of total RNA were rRNA-depleted using two rounds of the MICROBExpress™ Bacterial mRNA Enrichment Kit (Ambion). The efficiency of the depletion was evaluated in agarose gel 1% and all RNA preparations were quantified with a Nanodrop 1000 spectrophotometer. After rRNA depletion, 500 ng of depleted rRNA was used to construct the sequencing libraries following standard protocols using the SOLiD Total RNA-Seq Kit (Life Technologies). The libraries were barcoded by using the SOLiD Transcriptome Multiplexing Kit (Life Technologies). The emulsion PCR and SOLiD sequencing were performed according to standard Life Technologies protocols. Two independent samples were used to prepare replicate libraries resulting in a total of 4 libraries. Mapping of the reads against the *H. seropedicae* genome sequence, data processing and statistical analysis were performed using the CLC Genomics Workbench 5.1 and the results were expressed in RPKM (Reads Per Kilobase of exon model per Million mapped reads) (Mortazavi et al., 2008). The sequence data are available in the ArrayExpress database (www.ebi.ac.uk/arrayexpress) under accession number E-MTAB-3435.

A gene was considered expressed when read coverage was equal to or higher than 3-fold, and differentially expressed when RPKM value was 2-fold higher/smaller in +Nar compared to -Nar and *p*-value higher than 0.05 by the Baggerley's test as implemented in CLC Workbench. The Baggerley's test (Baggerly et al., 2003) compares the proportion of counts in a group of replicates (+Nar) against those of another group of replicates (−Nar), comprising a weighted *t*-type test statistic. The samples are given different weights depending on their sizes (total counts). The weights are obtained by assuming a Beta distribution on the proportions in a group, and estimating these, along with the proportion of a binomial distribution, by the method of moments. RNAseq statistical analyses were also made using the R package DESeq, which performs a negative binomial distribution and a shrinkage estimator for the distribution's variance and size-factor normalization (Anders and Huber, 2010).

#### Motility assay

The motility assay was performed on NFbHPN-malate semi-solid agar (0.25%) plates supplemented with 100 μM naringenin. Overnight culture of *H. seropedicae* was inoculated in the center of the plate and incubated at 30°C. The plates were photographed after 12 h and motility halos were measured by using ImageJ (Rasband, 1997). Ten replicates with 10^8^ bacteria were inoculated in both plates.

#### RT-qPCR

For validation with RT-qPCR, total RNA was isolated from cultures grown in the presence and absence of naringenin (100 μM) using the Ribominus (Ambion), the cDNAs were synthesized using the High-capacity cDNA Reverse Transcription kit (Applied Biosystems), and quantified using the Power SYBR-Green PCR Master Mix on a Step One Plus Real Time-PCR System (Applied Biosystems). The Primer express 3.0 software was used to design the primers. The 16S rRNA gene was used as internal control, and the relative gene expression was determined using the 2^−ΔΔCt^ method (Livak and Schmittgen, 2008).

#### Colonization assay

*H. seropedicae* SmR1 cells were grown in NFbHPN medium at 30°C, 120 rpm, until OD_600nm_ = 1. The culture was diluted to OD_600nm_ = 0.2 in fresh medium, and grown in the same conditions in the presence or absence of 100 μM naringenin for 6 h. These cells were washed with saline buffer and 10^5^ cells were inoculated per maize plantlet (samples in triplicate). Quantification of bacterial root endophytic populations was performed according to Balsanelli et al. (2013), every 12 h until 3 days after inoculation. Endophytic bacteria at time zero is too low to count (REF).

## Results and discussion

### Early *Herbaspirillum* seropedicae SmR1 maize root colonization is stimulated by naringenin

**Figure 1:**
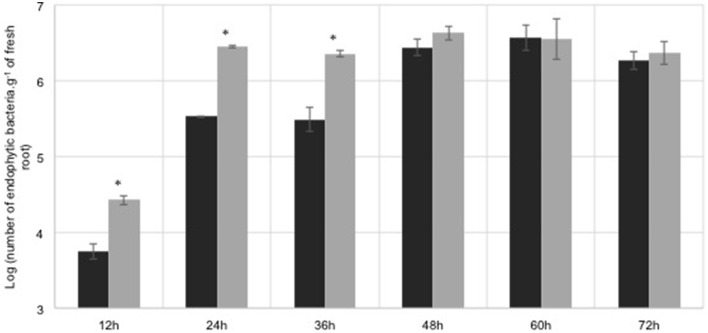
**Maize root endophytic colonization by** ***H. seropedicae*** **wild-type**. *H. seropedicae* SmR1 cells were grown in the presence (gray bars) or absence (black bars) of 100 μM naringenin for 6 h, and 10^5^ cells were inoculated on maize plantlets. The number of root endophytic bacteria was determined after the periods indicated. Results are shown as means of Log_10_ (number of endophytic bacteria.g^−1^ of fresh root) ± standard deviation. Asterisk indicates significant differences at *p* < 0.0083 (Student *t*-test with Bonferroni correction) of endophytic colonization between naringenin treated and non-treated bacteria.

Flavonoids constitute a large part of root exudates (Cesco et al., 2010), being involved in root colonization. Previously results showed that the plant-derived flavonoid naringenin regulated the expression of *H. seropedicae* genes. Maize roots were inoculated with *H. seropedicae* in the presence of naringenin (100 μM) to determine the effect of this flavonoid in colonization pattern. The results showed that the endophytic population in the first 36 h is higher in the presence of naringenin (Figure 1). After 36 h the endophytic population is the same in the absence and in the presence of naringenin. These results indicate that naringenin affects early endophytic colonization. Naringenin stimulation of root colonization was also observed during the colonization of *Arabidopsis thaliana* by *H. seropedicae* (Gough et al., 1997), and wheat by *Azorhizobium caulinodans* (Webster et al., 1998).

### Changes in the *Herbaspirillum seropedicae* SmR1 transcriptome in response to naringenin

RNA-seq profiling of *H. seropedicae* cells grown in the presence (+Nar) or absence (−Nar) of 100 μM of naringenin for 6 h was performed as described in Bacterial growth in Material and Methods. Sixty four million and fifty eight million reads were obtained for −Nar and +Nar conditions, respectively, and of those 2.7 million and 2.5 million were mapped uniquely to the *H. seropedicae* genome. As expected, biological replicates showed a very high level of correlation (*r*^2^ > 0.97) (Table 1), thus all the libraries of each condition were used for further analysis.

**Table 1:** **Summary of RNA-seq data**.

**Sample**	**Reads in biological replicates**	**Total reads**	**Reads mapped unambiguously^a^**	**Total reads mapped unambiguously replicates**	**Correlation (*R*^2^)**
−Naringenin 1	34,539,083	64,757,598	1,429,789	2,718,964	0.97
−Naringenin 2	30,218,515		1,289,175		
+Naringenin 1	14,525,262	58,754,928	683,731	2,478,145	0.98
+Naringenin 2	32,189,749		1,029,225		
+Naringenin 3	12,039,917		765,189		

a The reads were uniquely mapped to the H. seropedicae genome using CLC Genomics Workbench 5.1 will 90% of minimum length and 80% of similarity. The numbers 1 and 2 refer to biological replicates and the number 3 is technical replicate of the condition +Naringenin.

**Figure 2:**
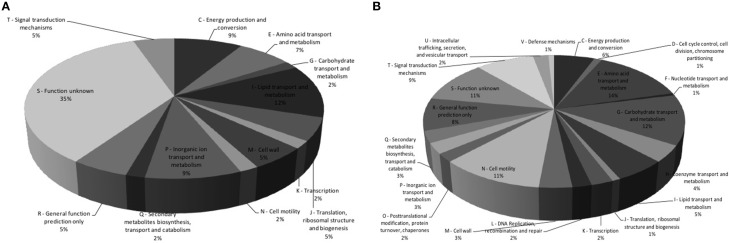
**Functional classification of genes upregulated and downregulated by naringenin**. Three hundred and four genes were upregulated **(A)** and 77 were downregulated **(B)** in the presence of naringenin. The genes were functional classificated by COG (Clusters if Orthologous Groups of proteins Tatusov et al., 1997) (http://www.ncbi.nlm.nih.gov/COG).

The genes that showed fold change greater than 2.0 (+Nar relative to −Nar) and a *p* = 0.05 were considered to be regulated by naringenin. Three hundred and four genes were downregulated and 77 were upregulated by naringenin by CLC Workbench. Fifty-three percent of these were also differentially regulated by the DESeq analysis (Table S1). The regulation of *flhB*, *flgE*, Hsero_2564 and *murF* genes are confirmed by qPCR (Table 2). Some genes with fold changes marginally lower than 2.0 fold or p higher than 0.05 were also considered regulated if neighborhood analysis suggested that they are part of an operon with genes regulated according to the previous criteria. The majority of the upregulated genes belong to the following functional gene categories: lipid transport and metabolism, energy production and conversion, inorganic ion transport and metabolism and unknown function (Figure 2A). The downregulated genes belong mainly to the categories aminoacid transport and metabolism, carbohydrate transport and metabolism, motility and unknown function (Figure 2B).

**Table 2:** **Genes differentially expressed in the presence of naringenin**.

**Gene**	**Fold Change Transcriptome^a^**	**qPCR^b^**
*flh*B	−2.65	0.599
*flg*E	−2.33	0.618
Hsero 2564	−7.37	0.476
*mur*F	−2.08	0.64

a The Fold change was determined by CLC Workbench 5.1.

b Relative expression in the presence of naringenin.

We found some groups of genes whose expression were regulated based on both CLC Workbench and DESeq analyses by naringenin that may be involved in plant-bacterial interaction. These genes are mainly involved with cell wall and motility. We also noticed an increase in the expression of genes that could be involved in naringenin degradation.

### *H. seropedicae* cell wall is altered in the presence of naringenin

**Figure 3:**
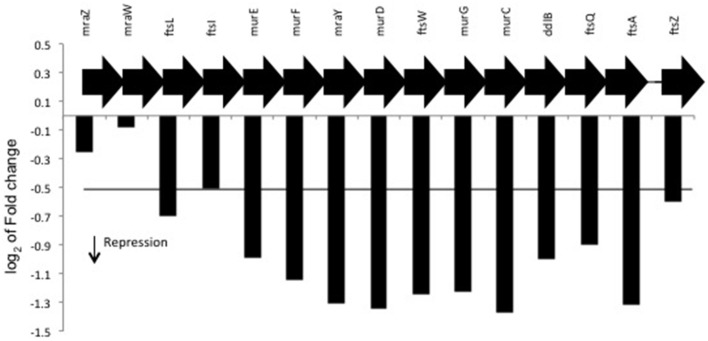
**Differential expression of region of** ***mur*** **genes in the presence of naringenin**. Region of *mur* genes in *H. seropedicae* genome. The value represents the log_2_ of fold change of genes.

Peptidoglycan is an essential component for synthesis of the bacterial cell wall and the biosynthesis of this molecule is a complex process that proceeds in several stages (Ramos et al., 2004; Patin et al., 2010; Muchova et al., 2011). The assembly of the peptidoglycan involves, the Mur ligases (MurC, MurD, MurE, and MurF) that catalyze the first step of the synthesis the UDP-N-acetylmuramoyl-pentapeptide precursor (Patin et al., 2010). Three genes that encode the enzymes MurC, MurD, and MurF were downregulated in the presence of naringenin (Figure 3). Other *mur* genes were also inhibited by naringenin such as genes encoding the enzymes MraY and MurG (Figure 3). MraY catalyzes the transfer of the phospho-N-acetyl-muramoyl-pentapeptide from UDP-N-acetyl-muramoyl-pentapeptide to a membrane acceptor to form lipid I. The final step of the peptidoglycan subunit biosynthesis is the addition of N-acetylglucosamine (GlcNAc) to lipid I catalyzed by MurG producing lipid II (Muchova et al., 2011).

Interestingly the genes *ddlB* and *ftsQAZ* were also downregulated by naringenin and found in the same operon as the *mur* genes (Figure 3, Figure S1A). D-Alanine–D-alanine ligase (coded by *ddlB*) is an enzyme involved in peptidoglycan biosynthesis and the proteins FtsQ, FtsA, and FtsZ are involved in septum formation in cell-division (Jofré et al., 2009). Mutations in *ddlB* and *ftsQAZ* genes in *Azospirillum brasilense* resulted in overproduction of exopolysaccharides, decreased bacterial tolerance to saline stress and alteration in cell morphology (Jofré et al., 2009).

A decrease in level of peptidoglycan synthesis enzyme GlmU was observed in the proteome of *H. seropedicae* grown in the presence of sugarcane extract (Cordeiro et al., 2013). In our study the expression of *glmU* was slightly decreased (−1.3-fold, *p* = 0) in the presence of naringenin, a decrease similar to that was observed by RT-PCR (-1.6-fold) in *H. seropedicae* grown in the presence of the sugarcane extract (Cordeiro et al., 2013). Peptidoglycan may act as elicitors of plant innate immunity, being recognized as a microbe-associated molecular pattern (MAMP) (Erbs and Newman, 2012). For example purified peptidoglycans from *Xanthomonas campestris* pv. *Campestris* and *Agrobacterium tumefaciens* act as MAMPs, inducing immune responses in *Arabidopsis thaliana*. The results suggest that the *H. seropedicae* is capable of controlling peptidoglycan synthesis in response to plant signals. A similar strategy has been described for *Listeria monocytogenes* that can N-deacetylate its peptidoglycan, avoiding the recognition and killing by host cells (Boneca et al., 2007). *Agrobacterium tumefaciens* also alters its peptidoglycan to reduce elicitation of plant defense (Erbs et al., 2008). Tadra-Sfeir et al. (2011) showed that the *ampG* gene of *H. seropedicae* is downregulated by naringenin), and the mutation in this gene alters the cell morphology. *ampG* codes for a muropeptide permease that is involved in the recycling of peptidoglycan. In the present transcriptome analysis the expression of this gene was slightly decreased (−1.2). It is possible that this difference is due to distinct growth condition used in the present work.

The genes *rfbG galE rfbBC* and *wcaGA* involved in lipopolyssacharide (LPS) biosynthesis were also downregulated by naringenin. Alterations in cell surface are common in other bacteria when they interact with plants or in the presence of plant compounds. *Rhizobium* sp. strain NGR234 synthesizes a new LPS in the presence of flavonoids and this LPS is important for the colonization of NGR234 in leguminous plants (Ardissone et al., 2011).

### Chemotaxis and flagella

Bacteria can sense the environment and rapidly respond to environmental changes through the action of specific signaling pathways. The chemotaxis signal begins with the binding of molecules on membrane receptors. Chemoreceptors are encoded by the *tsr, tar, trg* and *tap* genes, that code for methyl-accepting chemotaxis proteins (MCPs) (Pereira et al., 2004).

We identified forty-one genes involved in the chemotaxis transduction pathways in *Herbaspirillum seropedicae* genome. Twenty nine of these are found in five clusters and the other genes are monocistronics with 6 homologous to *cheA*, 5 to *cheB*, 10 to *cheD*, 5 to *cheR*, 1 to *cheM*, 9 to *cheY*, 1 to *cheZ*, and 5 to *cheW* (Pedrosa et al., 2011). Cluster I has five genes, *Hsero_0623* (methyl-accepting chemotaxis transducer transmembrane protein), *cheWRB* and the *Hsero_0627*. Cluster III of *H. seropedicae* contains *tar, cheRDBYZ* and *flhBA, three cheD*-like and eight genes coding for methyl-accepting chemotaxis proteins (*tsr*, Hsero_0538, 1262, 1556, 3234, 4019, 4543, and 4615) all these were repressed in the presence of naringenin. In contrast the *cheR* and *cheY* genes of the cluster II *cheWBRYA* were activated in the presence of the flavonoid. Cluster IV has *cheYAW* genes and the cluster V contains eight genes, one encoding a methyl-accepting chemotaxis protein (*Hsero_3022*), one a chemotaxis signal transduction protein (*Hsero_3021*), one a response regulator protein (*Hsero_3016*), one an acyl dehydratase protein (*Hsero_3015*), and the genes *cheRWAB*. The expression of the gene clusters I, IV, and V did not change in the presence of naringenin. In *Pseudomonas aeruginosa* the *che* genes are also organized in five gene clusters, which have different responses depending on the stimulus (Ferrandez et al., 2002; Guvener et al., 2006).

**Figure 4:**
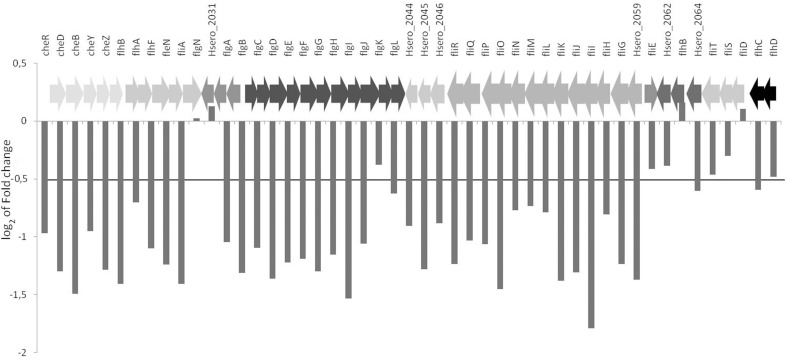
**Differential expression of flagellar genes in the presence of naringenin**. The value represents the log_2_ of fold change of genes. Genes of the same color are in the same operons.

In bacteria, flagella genes are regulated in response to environmental changes. These genes are found in operons that are divided in three temporally regulated transcriptional classes: early (class 1), middle (class 2), and late (class 3) (Komeda, 1986; Kutsukake et al., 1994). *H. seropedicae* has at least 46 genes involved in flagella biosynthesis, assembly and structure. *H. seropedicae* early genes homologous to the class 1 *flhC* and *flhD*, to middle genes class 2 *flgA*, *flgBCDEFGHI*, *flhB*, *fliA*, *fliD*, *fliFGHIJK*, *fliOPQR*, and late genes of class 3 *tsr*, *cheA*, *cheRBYZ* are all repressed in the presence of naringenin (Figure 4, Figure S1B). The FlhCD proteins are sigma 70 dependent transcriptional activators of class 2 promoters (Kutsukake et al., 1994; Liu and Matsumura, 1994) and FliA protein is a flagella alternative sigma factor – σ^28^ (Ohnishi et al., 1990). FliA has been shown to be involved in transcription of flagella, chemotaxis, and motility genes and the decrease in the expression of this protein could be responsible for the decrease in expression of middle and late flagella genes and chemotaxis genes (Iriarte et al., 1995).

Naringenin regulates flagellar genes expression in other bacteria. The transcriptome profile of *Salmonella typhimurium* LT2 revealed that 24 genes of pathogenicity island 1 and 17 genes involved in flagellar and motility were repressed in the presence of naringenin (Vikram et al., 2011). Flagellar genes were also downregulated in *A. caulinodans* by naringenin (Tsukada et al., 2009) and *Pseudomonas syringae* pv. tomato DC3000 by phloretin (Vargas et al., 2013). In *Bacillus subtilis* OKB105 chemotaxis and motility genes were downregulated in response to rice seedlings (Xie et al., 2015), suggesting that decrease of motility in the presence of root exudate maybe be involved in the establishment of interaction with the plant. Signal molecules released by plants direct the bacteria toward the root in a process dependent on chemotaxis and cell motility. At this initial stage the concentration of signal compounds are low and the bacteria follow a concentration gradient and bacteria motility depends on flagella-driven motility. Upon reaching root surface the bacteria would attach and reduce flagella gene expression and motility may depend on other means more appropriate for this new environment, such as type IV *pilli*-dependent swarming. Alternatively flavonoids such as naringenin acting as an anti-microbial agent targets the flagella, as suggested for *P. syringae* (Vargas et al., 2013).

**Figure 5:**
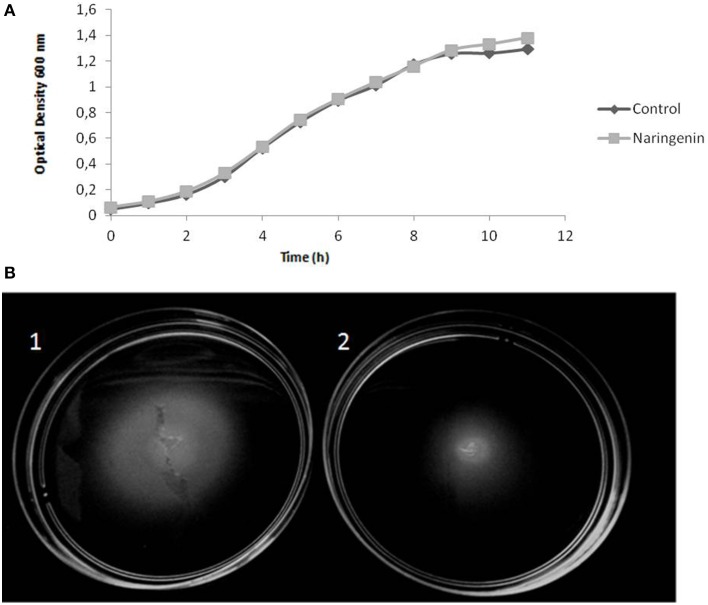
**Motility of** ***H. seropedicae*** **SmR1 is reduced by the presence of naringenin**. **(A)** Growth curve of *H. seropedicae* SmR1 in liquid NFbHPN-malate. **(B)** Motility of *H. seropedicae* SmR1 in semi-solid NFbHPN-malate in the absence (1) or presence (2) of 100 μM naringenin. Growth halos in semi-solid medium of ten replicates were analyzed by ImageJ software. Control = 1.00 ± 0.15; with naringenin = 0.70 ± 0.14, the values are arbitrary units in relation to the mean of the control condition.

Downregulation of flagella and chemotaxis genes indicated that naringenin might reduce *H. seropedicae* motility. This hypothesis was tested by measuring the motility of *H. seropedicae* in the presence of naringenin. The results demonstrated that naringenin impairs *H. seropedicae* motility (Figure 5B) without affecting the growth in liquid medium (Figure 5A).

### Aromatic compounds metabolism is induced by naringenin

*H. seropedicae* genome sequence analysis showed the existence of genes involved in aromatic compounds metabolism such as catechol (*cat*), benzoate (*ben*), 4-hydroxybenzoate (*pob*), phenylacetate (*paa*), and protocatechuate (*pca*) metabolism (Pedrosa et al., 2011). These compounds can be metabolized to tricarboxylic acid intermediates.

In *H. seropedicae* the expression of *pcaJIF* and *catCD* genes increased by 3 -7 fold in the presence of naringenin, these genes are involved in the conversion of muconolactone to 3-oxoadipyl-Coa a pathway of the catechol metabolism. The expression of *paaBC* and *Hsero_4130* also increased in the presence of naringenin. These three genes are probably organized in an operon with other *paa* genes. The *paa*, *pca*, and *cat* genes may be involved in naringenin intermediates degradation.

Another important system influenced by naringenin treatment was the multidrug efflux. *H. seropedicae* genome has five regions containing genes *acrAB;* these regions were named Cluster I, II, III, IV, and V. In this study, *acrA* and *acrB* of the cluster II were induced 7.5 and 3.2-fold, respectively. In *S. typhimurium* LT2 the genes *acrAB* were also induced 3-fold in the presence of naringenin (Vikram et al., 2010). Other genes involved in multidrug transport efflux were also induced by naringenin such as *ompC* (2.4-fold) and *Hsero_1358* (3.15-fold). Multidrug transporter efflux pump provides low level of resistance to alkaline dyes, detergents and antibiotics. Induction of this efflux pump by naringenin suggests activation of the drug resistance system.

We found 35 ABC-transporter gene clusters differentially expressed in the presence of naringenin, being 29 downregulated and 6 upregulated (Table S1). The main group downregulated are amino acid and sugar transporter with a putative aromatic amino acid transporter system (~7-fold down-regulated) and a sugar transporter repressed 4-fold. Among the upregulated ABC transporter we found mainly anion transporters such as sulfate, phosphate and alkanesulfanates. A proteomics approach revealed that *Bradyrhizobium japonicum* strains differentially expressed three periplasmic amino acid binding proteins of ABC-transporter systems in the presence of genistein (Batista and Hungria, 2012). The reason for this effect is not known.

In this study we provided a comprehensive view of a *H. seropedicae* transcriptome in the presence of naringenin. We have defined in detail the RNA populations found in *H. seropedicae* in the presence and absence of the flavonoid. The data obtained from this study enabled us to infer some aspects of the metabolism of the bacteria in the presence of naringenin. Expression of genes related to bacterial flagella biosynthesis, flagella motor activity, and chemotaxis were repressed by naringenin, and this repression is predicted to have negative effects on flagella synthesis and bacterial motility. Biosynthesis of peptidoglycan is also inhibited by narigenin, whereas expression of a multidrug transport efflux pump is activated. The data suggest that in the presence of naringenin *H. seropedicae* triggers a concerted change in gene expression probably related to defense mechanisms.

### Conflict of interest statement

The authors declare that the research was conducted in the absence of any commercial or financial relationships that could be construed as a potential conflict of interest.
