# Supplementary material for: Genome wide transcriptional profiling of Herbaspirillum seropedicae SmR1 grown in the presence of naringenin
Source: Front Microbiol. 2015 May 21;6:491. doi: 10.3389/fmicb.2015.00491 (PMC4440368; doi:10.3389/fmicb.2015.00491)
Supplement: Supplementary file 1 [file Table1.DOC]

| **Gene** | **Predict Function** | **Fold Change** | **p Value** |
| --- | --- | --- | --- |
| Hsero_0014 | conserved hypothetical protein | -2.03 | 0.02 |
| Hsero_0046 ^#^ | major facilitator superfamily (MFS) transporter transmembrane protein | 2.38 | 0.02 |
| *glnH* | ABC-type glutamine transport system. periplasmic component protein | -2.06 | 0.00 |
| *glnP ** | ABC-type glutamine transport system. permease component protein | -1.29 | 0.15 |
| *glnQ* | ABC-type glutamine transport system. ATPase component protein | -2.15 | 0.01 |
| Hsero_0083 ^#^ | membrane protein | 3.25 | 0.00 |
| *glnK ^#^* | nitrogen regulatory PII-like protein | 3.53 | 0.05 |
| *amtB*^#^* | ammonium transporter transmembrane protein | 2.89 | 0.06 |
| *rplL ^#^* | 50s ribosomal subunit L7/L12 protein | 2.86 | 0.00 |
| *rplN** | 50S ribosomal subunit L14 protein | 1.58 | 0.25 |
| *rplX** | 50S ribosomal subunit L24 protein | 1.73 | 0.13 |
| *rplE** | 50S ribosomal subunit L5 protein | 1.86 | 0.17 |
| *rpsN** | 30S ribosomal subunit S14 protein | 1.48 | 0.22 |
| *rpsH** | 30S ribosomal subunit S8 protein | 1.82 | 0.07 |
| *rplF** | 50S ribosomal subunit L6 protein | 1.57 | 0.26 |
| *rplR** | 50S ribosomal subunit L18 protein | 1.80 | 0.11 |
| *rpsE** | 30S ribosomal subunit S5 protein | 1.74 | 0.14 |
| *rpmD ^#^* | 50S ribosomal subunit L30 protein | 2.76 | 0.00 |
| *rplO** | 50S ribosomal subunit L15 protein | 1.66 | 0.33 |
| *secY*** | preprotein translocase SecY transmembrane protein | 2.18 | 0.06 |
| *infA ^#^* | translation initiation factor (IF-1) protein | 7.32 | 0.01 |
| *rpmJ* | 50S ribosomal subunit L36 protein | 2.40 | 0.00 |
| *rpsM*** | 30S ribosomal subunit S13 protein | 2.16 | 0.07 |
| *rpsK ^#^* | 30S ribosomal subunit S11 protein | 2.35 | 0.04 |
| Hsero_0148 | ABC-type branched-chain amino acid transport system. periplasmic component protein | -2.12 | 0.00 |
| Hsero_0253* | transcription regulator protein | -1.79 | 0.00 |
| Hsero_0254 | acyl-CoA dehydrogenase protein | -2.02 | 0.00 |
| Hsero_0300 ^#^ | aldo/keto reductase protein | -2.76 | 0.00 |
| Hsero_0310 | HD-GYP domain containing protein | -2.06 | 0.00 |
| Hsero_0311 | transmembrane protein | -2.00 | 0.01 |
| *hpcH* | 2.4-dihydroxyhept-2-ene-1.7-dioic acid aldolase | -2.98 | 0.00 |
| *mraZ** | cell division protein | -1.15 | 0.73 |
| *mraW** | S-adenosyl-methionine-dependent methyltransferase | 1.06 | 0.59 |
| *ftsL** | cell division | -1.62 | 0.18 |
| *ftsI** | cell division | -1.33 | 0.11 |
| *murE** | UDP-N-acetylmuramoylalanyl-D-glutamate-2.6- diaminopimelate ligase | -1.81 | 0.06 |
| *murF* | UDP-N-acetylmuramoyl-tripeptide-D-alanyl-D- alanine ligase protein | -2.08 | 0.00 |
| *mraY ^#^* | UDP-N-acetylmuramyl pentapeptide phosphotransferase/UDP-N- acetylglucosamine-1-phosphate transferase protein | -2.36 | 0.01 |
| *murD ^#^* | UDP-N-acetylmuramoylalanine-D-glutamate ligase protein | -2.43 | 0.00 |
| *ftsW* | rod shape-determining (RodA protein) transmembrane protein | -2.26 | 0.00 |
| *murG* | UDP-N-acetylglucosamine-N-acetylmuramyl- pentapeptide pyrophosphoryl-undecaprenol N-acetylglucosamine transferase protein | -2.34 | 0.02 |
| *murC* | UDP-N-acetylmuramate-L-alanine ligase protein | -2.43 | 0.00 |
| *ddlB* | D-alanine-D-alanine ligase B protein | -2.00 | 0.00 |
| *ftsQ* | cell division septal protein | -2.00 | 0.00 |
| *ftsA ^#^* | actin-like ATPase involved in cell division protein | -2.37 | 0.00 |
| Hsero_0348* | lysophospholipase protein | -1.55 | 0.61 |
| *argJ** | N-acetylglutamate synthase protein | -1.41 | 0.21 |
| Hsero_0350* | AAA+ superfamily ATPase protein | -1.65 | 0.02 |
| *mutT* | pyrophosphohydrolase protein | -2.21 | 0.00 |
| *corB* | Mg2+/Co2+ transporter transmembrane protein | -2.00 | 0.00 |
| Hsero_0359* | hypothetical protein | -1.08 | 0.52 |
| Hsero_0440 ^#^ | ABC-type sugar transport system. periplasmic component protein | -2.91 | 0.00 |
| Hsero_0441 | ABC-type sugar transport system. permease component protein | -2.01 | 0.00 |
| Hsero_0442 | ABC-type sugar transport system. permease component protein | -2.43 | 0.01 |
| Hsero_0443 ^#^ | phosphatase/phosphohexomutase protein | -3.07 | 0.00 |
| Hsero_0444 ^#^ | ABC-type sugar transport system. ATPase component protein | -2.56 | 0.00 |
| Hsero_0516 | pyrophosphatase protein | -2.06 | 0.05 |
| Hsero_0517* | SAM-dependent methyltransferase protein | -1.60 | 0.04 |
| Hsero_0522 | lactoylglutathione lyase-related protein | 3.46 | 0.00 |
| Hsero_0538 | methyl-accepting chemotaxis transducer transmembrane protein | -2.05 | 0.00 |
| Hsero_0556 | hypothetical protein | 3.67 | 0.00 |
| Hsero_0585 ^#^ | 2.4'-dihydroxyacetophenone dioxygenase protein | -3.18 | 0.00 |
| Hsero_0623* | methyl-accepting chemotaxis transducer transmembrane protein | -1.17 | 0.53 |
| *cheW** | positive regulator of CheA protein activity protein | 1.54 | 0.24 |
| *cheR* | cheR chemotaxis protein methyltransferase protein | 2.49 | 0.00 |
| *cheB ** | chemotaxis-specific methylesterase protein | 1.29 | 0.52 |
| Hsero_0627* | response regulator protein | 1.04 | 0.98 |
| Hsero_0643 ^#^ | membrane transport protein | -3.67 | 0.00 |
| Hsero_0644 ^#^ | conserved hypothetical protein | -4.18 | 0.00 |
| Hsero_0648 ^#^ | transmembrane protein | -3.49 | 0.00 |
| *azlC ^#^* | branched-chain amino acid permease (azaleucin resistance) protein | -2.39 | 0.00 |
| *ompW2* | outer membrane W protein | -2.08 | 0.00 |
| *pepP ^#^* | XAA-PRO aminopeptidase protein | -2.54 | 0.00 |
| *gloA* | lactoylglutathione lyase protein | -2.44 | 0.00 |
| *eriC ** | chloride-channel protein | -1.69 | 0.47 |
| *lnt** | apolipoprotein N-acyltransferase transmembrane protein | -1.67 | 0.11 |
| *corC* | Mg2+ and Co2+ transporter protein | -2.39 | 0.00 |
| Hsero_0727 | ABC-type sugar transport system. periplasmic component protein | -2.09 | 0.02 |
| Hsero_0728* | ABC-type sugar transport system. ATPase component protein | -1.70 | 0.01 |
| Hsero_0729 | ABC-type sugar transport system. permease component protein | -2.16 | 0.02 |
| Hsero_0730 ^#^ | N-acyl-D-glucosamine 2-epimerase (AGE domain) protein | -2.44 | 0.00 |
| Hsero_0898 | hypothetical protein | 6.39 | 0.02 |
| Hsero_0899* | bacteriophage tail completion-like protein | 1.56 | 0.28 |
| *acs ** | acetyl-coenzyme A synthetase protein | 1.83 | 0.00 |
| *Hsero_0925 ^#^* | 3-hydroxyacyl-CoA (short-chain) dehydrogenase type II oxidoreductase protein | 3.55 | 0.01 |
| *atoB ^#^* | acetyl-CoA acetyltransferase (acetoacetyl-CoA thiolase) protein | 3.31 | 0.00 |
| Hsero_0927 ^#^ | acyl-CoA dehydrogenase oxidoreductase protein | 3.45 | 0.00 |
| Hsero_0946 | HD-GYP domain containing protein | -2.01 | 0.02 |
| *glpD2** | glycerol-3-phosphate dehydrogenase protein | -1.41 | 0.06 |
| Hsero_0967 ^#^ | ABC-type sugar transport system. ATPase component protein | -2.17 | 0.00 |
| Hsero_0968* | ABC-type sugar transport system. ATPase component protein | -1.50 | 0.09 |
| Hsero_0969* | ABC-type sugar transport system. permease component protein | -1.53 | 0.00 |
| Hsero_0970 | ABC-type sugar transport system. permease component protein | -2.15 | 0.00 |
| Hsero_0971* | integral transmembrane protein | -1.28 | 0.37 |
| Hsero_0972 ^#^ | ABC-type sugar transport system. periplasmic component protein | -2.81 | 0.00 |
| *aidB ^#^* | acyl-CoA dehydrogenase protein | -2.23 | 0.00 |
| Hsero_1029 ^#^ | galactonate dehydratase | -3.07 | 0.00 |
| Hsero_1033 ^#^ | ABC-type sugar transport system. periplasmic component protein | -3.98 | 0.00 |
| Hsero_1034 ^#^ | ABC-type sugar transport system. ATPase component protein | -2.57 | 0.00 |
| Hsero_1035 ^#^ | ABC-type sugar transport system. permease component protein | -4.63 | 0.00 |
| Hsero_1036 ^#^ | ABC-type sugar transport system. permease component protein | -5.12 | 0.00 |
| Hsero_1037 ^#^ | ABC-type sugar transport system. periplasmic component protein | -3.00 | 0.00 |
| Hsero_1038* | ABC-type sugar transport system. ATPase component protein | -1.81 | 0.00 |
| Hsero_1039 | ABC-type sugar transport system. permease protein | -2.17 | 0.00 |
| Hsero_1040 | dihydroxyacid dehydratase/phosphogluconate dehydratase protein | -2.17 | 0.00 |
| Hsero_1041 | dehydrogenase oxidoreductase protein | -2.13 | 0.03 |
| Hsero_1043 ^#^ | outer membrane (porin) protein | -6.42 | 0.00 |
| Hsero_1044 ^#^ | gluconolactonase protein | -2.97 | 0.01 |
| Hsero_1046 | conserved hypothetical protein | -2.07 | 0.00 |
| Hsero_1047* | short-chain alcohol dehydrogenase protein | -1.60 | 0.00 |
| Hsero_1048* | conserved hypothetical protein | -1.62 | 0.00 |
| Hsero_1049 | ABC-type sugar transport system. ATPase component protein | -2.00 | 0.00 |
| Hsero_1050 ^#^ | ABC-type sugar transport system. permease component protein | -2.39 | 0.00 |
| Hsero_1051 ^#^ | ABC-type sugar transport system. periplasmic component protein | -2.63 | 0.00 |
| *metX* | homoserine acetyltransferase protein | -2.00 | 0.01 |
| Hsero_1070 | conserved hypothetical protein | -2.20 | 0.00 |
| *potD ^#^* | ABC-type spermidine/putrescine transport system. periplasmic protein protein | -3.62 | 0.00 |
| Hsero_1085 | hemolysin protein | -2.16 | 0.02 |
| *ggt* | gamma-glutamyltranspeptidase protein | -2.76 | 0.00 |
| *talB* | transaldolase protein | -2.05 | 0.02 |
| Hsero_1097* | transcription regulator protein | -1.59 | 0.00 |
| *zwf ^#^* | glucose-6-phosphate 1-dehydrogenase protein | -2.29 | 0.00 |
| *pgi ^#^* | glucose-6-phosphate isomerase protein | -2.03 | 0.01 |
| *edd** | 6-phosphogluconate dehydratase protein | -1.79 | 0.00 |
| *eda ^#^* | 2-keto-4-hydroxyglutarate aldolase/-keto-3-deoxy-6-phosphogluconate aldolase protein | -2.27 | 0.00 |
| Hsero_1130 ^#^ | ABC-type dipeptide transporter. periplasmic peptide-binding protein | -2.99 | 0.00 |
| Hsero_1134 ^#^ | enzyme od the cupin superfamily protein | -6.16 | 0.00 |
| *dadA ** | Glycine/D-amino acid oxidases (deaminating) protein | -1.51 | 0.17 |
| *maeB* | NADP-dependent malic enzyme oxidoreductase protein | -2.02 | 0.01 |
| Hsero_1173* | 3-hydroxyacyl-CoA dehydrogenase type II oxidoreductase protein | -1.26 | 0.15 |
| Hsero_1174 | esterase of the alpha-beta hydrolase superfamily protein | -1.86 | 0.00 |
| *elaB ^#^* | transmembrane protein | 3.80 | 0.00 |
| Hsero_1196 | conserved hypothetical protein | 3.12 | 0.00 |
| Hsero_1205 | hypothetical protein | -2.01 | 0.00 |
| *cheD ^#^* | methyl-accepting chemotaxis I (serine chemoreceptor) transmembrane protein | -2.06 | 0.00 |
| *pstS ^#^* | ABC-type phosphate transport system. periplasmic component protein | 2.76 | 0.04 |
| Hsero_1262 ^#^ | methyl-accepting chemotaxis protein I | -2.43 | 0.00 |
| Hsero_1268 ^#^ | acyl-CoA transferases/carnitine dehydratase protein | 3.42 | 0.00 |
| Hsero_1271 ^#^ | conserved hypothetical protein 1442586:1443461 forward | -2.86 | 0.00 |
| *nahD ** | 2-hydroxychromene-2-carboxylate isomerase protein | -1.56 | 0.00 |
| *catD ^#^* | 3-ketoadipate enol-lactone hydrolase protein | 3.20 | 0.02 |
| *catC* | muconolactone delta-isomerase protein | 6.87 | 0.00 |
| Hsero_1333 | monooxygenase FAD-binding protein | -2.06 | 0.02 |
| Hsero_1337 | two component response regulator protein | -2.15 | 0.01 |
| Hsero_1356 ^#^ | transcription regulator protein | 4.97 | 0.03 |
| Hsero_1357 ^#^ | efflux transporter. RND family. MFP subunit protein | 7.50 | 0.00 |
| Hsero_1358 ^#^ | cation/multidrug efflux pump protein | 3.15 | 0.03 |
| *tolC ** | outer membrane drug efflux lipoprotein | 1.65 | 0.49 |
| Hsero_1360* | Hsero_1360 transcription regulator protein | -1.48 | 0.56 |
| Hsero_1369* | LysR family transcription regulator protein | -1.06 | 0.83 |
| Hsero_1370 | transcription regulator protein | -2.23 | 0.00 |
| Hsero_1371 ^#^ | transcription regulator protein | 2.73 | 0.00 |
| *glnE* | glutamate-ammonia-ligase adenylyltransferase (glutamine-synthetase adenylyltransferase) protein | -2.17 | 0.00 |
| *ansB* | L-asparaginase/archaeal Glu-tRNAGln amidotransferase subunit D protein | -2.27 | 0.00 |
| *aceE* | pyruvate dehydrogenase E1 component protein | -2.11 | 0.00 |
| *aceF* | dihydrolipoamide acetyltransferase component of pyruvate dehydrogenase complex (E2) protein | -2.05 | 0.00 |
| *lpdA ** | 2-oxoglutarate dehydrogenase. E3 component. lipoamide dehydrogenase protein | -1.80 | 0.00 |
| *fdsG* | NAD-dependent formate dehydrogenase gamma subunit protein | -2.14 | 0.00 |
| *fdsB ^#^* | NAD-dependent formate dehydrogenase beta subunit protein | -2.35 | 0.00 |
| *fdhF** | NAD-dependent formate dehydrogenase. alpha subunit. protein | -1.65 | 0.01 |
| *fdsC** | fdsC formate dehydrogenase regulatory subunit protein | -1.56 | 0.10 |
| Hsero_1496 | ABC-type amino acid transport system. permease component protein | -2.01 | 0.04 |
| Hsero_1497* | ABC-type amino acid transport system. permease component protein | -1.43 | 0.29 |
| Hsero_1498* | ABC-type amino acid transport system. ATPase component protein | -1.23 | 0.35 |
| Hsero_1499* | sugar phosphate isomerase/epimerase protein | -1.79 | 0.14 |
| Hsero_1500* | 2-ketogluconate kinase protein | -1.73 | 0.09 |
| Hsero_1552** | conserved hypothetical protein | 1.72 | 0.25 |
| Hsero_1553 ^#^ | 2-polyprenyl-6-methoxyphenol hydroxylase protein | 2.75 | 0.04 |
| Hsero_1554 ^#^ | hypothetical protein | -4.44 | 0.00 |
| *cheY ^#^* | chemotaxis regulator transmits chemoreceptor signals to flagellar motor components protein | -1.09 | 0.00 |
| Hsero_1556 | methyl-accepting chemotaxis transducer transmembrane I protein | -2.27 | 0.00 |
| Hsero_1610 | conserved hypothetical protein | -1.86 | 0.00 |
| *rimL ** | acetyltransferase protein | -1.59 | 0.25 |
| *moxR** | ATPase protein | -1.76 | 0.00 |
| Hsero_1614 | site-specific recombinase transmembrane protein | -2.06 | 0.00 |
| Hsero_1616* | periplasmic cytochrome c553 protein | -1.32 | 0.00 |
| Hsero_1617 ^#^ | periplasmic cytochrome c553 protein | -2.52 | 0.00 |
| *arsB** | NA+/H+ antiporter permease protein | -1.49 | 0.01 |
| Hsero_1631 | dehydrogenase oxidoreductase protein | -2.14 | 0.00 |
| *moeA* | moeA molybdopterin biosynthesis enzyme protein | -2.14 | 0.00 |
| *sbp*** | ABC-type sulfate transport system. periplasmic component protein | 2.74 | 0.21 |
| Hsero_1647** | NAD(P)H-dependent FMN reductase protein | 2.41 | 0.23 |
| *ssuA *** | ABC-type alkanesulfonates transport system. periplasmic component protein | 2.64 | 0.31 |
| *ssuD*** | alkanesulfonate monooxygenase protein | 3.39 | 0.16 |
| *ssuC*** | ABC-type alkanesulfonates transport system. permease component protein | 2.20 | 0.24 |
| *ssuB**^#^* | ABC-type alkanesulfonates transport system. ATPase component protein | 2.56 | 0.12 |
| *ssuF ^#^* | molybdopterin-binding protein | 6.05 | 0.00 |
| Hsero_1653**^#^ | EAL domain containing protein | 4.13 | 0.06 |
| Hsero_1654** | alpha/beta hydrolase fold esterase | 1.75 | 0.52 |
| *livK ** | ABC-type branched-chain amino acid transport system. periplasmic component protein | 1.16 | 0.72 |
| *livH* | ABC-type branched-chain aminoacid transport system. permease component protein | -2.19 | 0.01 |
| *livM** | ABC-type branched-chain amino acid transport system. permease component protein | -1.50 | 0.09 |
| *livG ** | ABC-type branched-chain amino acid transport system. ATPase component protein | -1.18 | 0.37 |
| *livF ** | ABC-type branched-chain amino acid transport system. ATPase component protein | -1.75 | 0.00 |
| Hsero_1693 | hypothetical protein | 2.44 | 0.00 |
| Hsero_1694 | GGDEF family protein | -2.13 | 0.01 |
| *cheY* | chemotaxis response regulator receiver domain protein | 2.26 | 0.02 |
| Hsero_1698* | methyl-accepting chemotaxis protein | 1.87 | 0.00 |
| *cheW ** | chemotaxis signal transduction protein | 1.45 | 0.23 |
| *cheB ** | cheB protein-glutamate methylesterase protein | 1.21 | 0.52 |
| Hsero_1701* | HEAT repeat protein | 1.58 | 0.14 |
| *cheR* | chemotaxis protein methyltransferase protein | 2.00 | 0.00 |
| *cheY* | chemotaxis CheY protein | 2.50 | 0.01 |
| *cheA ** | chemotaxis histidine kinase protein | 1.66 | 0.00 |
| Hsero_1711* | permease of the major facilitator superfamily protein | -1.37 | 0.32 |
| Hsero_1712 ^#^ | conserved hypothetical protein | -2.24 | 0.00 |
| *opuBB1** | ABC-type proline/glycine betaine transport system. permease component protein | 1.76 | 0.00 |
| *opuBC** | ABC-type proline/glycine betaine transport system. periplasmic binding component protein | 1.40 | 0.28 |
| *opuBB2* | ABC-type proline/glycine betaine transport systems. permease component protein | 2.66 | 0.00 |
| *opuA** | ABC-type proline/glycine betaine transport system. ATPase component protein | 1.99 | 0.01 |
| Hsero_1721 | esterase_lipase superfamily protein | -2.31 | 0.01 |
| Hsero_1722 | conserved hypothetical protein | -1.33 | 0.23 |
| *suhB** | inositol monophosphatase (extragenic suppressor) protein | -1.02 | 0.95 |
| *etf* | electron transfer flavoprotein-ubiquinone oxidoreductase protein | -2.09 | 0.00 |
| Hsero_1784 ^#^ | Hsero_1784 thioesterase protein | -3.30 | 0.00 |
| *livK* | ABC-type branched-chain amino acid transport system. periplasmic component protein | -2.21 | 0.01 |
| *livH* | ABC-type branched-chain amino acid transport system. permease component protein | -2.04 | 0.00 |
| *livG ^#^* | ABC-type branched-chain amino acid transport system. ATPase component protein | -2.76 | 0.00 |
| *livF* | ABC-type branched-chain amino acid transport system. ATPase component protein | -2.56 | 0.00 |
| Hsero_1795 | G:T/U mismatch-specific DNA glycosylase protein | -2.10 | 0.00 |
| Hsero_1805* | conserved hypothetical protein | -1.43 | 0.01 |
| Hsero_1806 ^#^ | ABC-type bacteriocin/antibiotic ATPase/permease fusion exporter protein | -3.04 | 0.00 |
| Hsero_1807 ^#^ | hemolysin-adenylate cyclase protein | -2.81 | 0.00 |
| Hsero_1823 ^#^ | hypothetical protein | 7.93 | 0.00 |
| *parC** | DNA topoisomerase IV (subunit A) protein | -1.99 | 0.00 |
| Hsero_1834* | transglycosylase signal peptide protein | -1.48 | 0.03 |
| *parE* | DNA topoisomerase IV subunit B protein | -2.03 | 0.01 |
| *ddpA* | ABC-type dipeptide transport system. periplasmic protein | -2.01 | 0.00 |
| Hsero_1848* | conserved hypothetical protein | 1.42 | 0.38 |
| Hsero_1849 | conserved hypothetical protein | 2.47 | 0.00 |
| *trnL* | tRNA-Leu | -2.60 | 0.00 |
| *moeA* | molybdopterin biosynthesis MoeA protein | -2.36 | 0.01 |
| *mobA** | molybdopterin-guanine dinucleotide biosynthesis protein A | -1.41 | 0.15 |
| *moaA ** | molybdenum cofactor biosynthesis protein A | -1.42 | 0.00 |
| *exaC ^#^* | NAD+ dependent acetaldehyde dehydrogenase protein | 3.84 | 0.00 |
| Hsero_1897 ^#^ | Hsero_1897 conserved hypothetical | 2.99 | 0.00 |
| Hsero_1898 | sigma-54 dependent transcription activator protein | -2.10 | 0.00 |
| *recO* | DNA repair protein | -2.33 | 0.00 |
| *pdxJ** | pyridoxal phosphate biosynthetic protein | -1.45 | 0.10 |
| *acpS** | phosphopantetheinyl transferase (holo-ACP synthase) protein | -1.06 | 0.47 |
| Hsero_1941* | beta-N-acetylhexosaminidase protein | -1.65 | 0.05 |
| *uvrC* | excinuclease ABC subunit C (SOS response DNA repair protein) protein | -2.04 | 0.00 |
| *epsL** | EPS biosynthesis protein | 1.84 | 0.24 |
| *epsB*^#^* | glucosyltransferase involved in lipopolysaccharide synthesis protein | 1.89 | 0.12 |
| *epsD** | peptidyl/prolyl cis-trans isomerase protein | -1.05 | 0.81 |
| *epsA** | periplasmic polysaccharide export protein | 1.32 | 0.60 |
| *epsF ^#^* | exopolysaccharide biosynthesis protein | 2.59 | 0.00 |
| *epsG** | EPS biosynthesis protein | 1.09 | 0.95 |
| *epsH** | exopolysaccharide methanolan synthase protein | 1.06 | 0.89 |
| *epsM** | cholera toxin secretion EpsM protein | -1.19 | 0.46 |
| Hsero_1995* | glycosyl transferase group 1 family protein | 1.94 | 0.13 |
| Hsero_1996* | acyltransferase protein | 3.38 | 0.10 |
| Hsero_1997* | O-antigen acetylase protein | 1.01 | 0.95 |
| *cheR* | chemotaxis protein methyltransferase | -2.09 | 0.00 |
| *cheD ^#^* | chemotaxis protein | -2.46 | 0.00 |
| *cheB ^#^* | chemotaxis-specific methylesterase protein | -2.96 | 0.00 |
| *cheY ** | chemotaxis protein | -1.97 | 0.01 |
| *cheZ ^#^* | chemotaxis phosphatase. CheZ protein | -2.44 | 0.00 |
| *flhB ^#^* | flagellar biosynthesis FlhB transmembrane protein | -2.65 | 0.00 |
| *flhA** | flagellar biosynthesis FlhA transmembrane protein | -1.63 | 0.00 |
| *flhF* | flagellar biosynthesis GTP-binding protein | -2.14 | 0.00 |
| *fleN ^#^* | antiactivator of flagellar biosynthesis FleN protein | -2.37 | 0.00 |
| *fliA ^#^* | RNA polymerase sigma factor for flagellar operon (sigma-F factor) protein | -2.65 | 0.00 |
| *flgA* | flagellar basal body P-ring formation protein | -2.06 | 0.03 |
| *flgB ^#^* | flagellar basal-body rod FlgB protein | -2.48 | 0.00 |
| *flgC* | flagellar basal-body rod protein | -2.14 | 0.00 |
| *flgD ^#^* | flagellar basal-body rod modification protein | -2.57 | 0.00 |
| *flgE ^#^* | flagellar basal-body rod FlgE protein | -2.33 | 0.00 |
| *flgF ^#^* | flagellar basal body rod FlgF protein | -2.28 | 0.00 |
| *flgG ^#^* | flagellar basal-body rod FlgG protein | -2.46 | 0.00 |
| *flgH* | flagellar basal-body L-ring protein | -2.23 | 0.00 |
| *flgI ^#^* | flagellar basal-body P-ring protein | -2.89 | 0.00 |
| *flgJ* | flagellar basal-body FlgJ protein | -2.08 | 0.00 |
| *flgK** | flagellar hook-associated FlgK protein | -1.30 | 0.24 |
| *flgL** | flagellar hook-filament junction protein. N-terminus flagellin protein | -1.54 | 0.00 |
| *fliR* | FliR component of the flagellar biosynthesis pathway | -2.36 | 0.00 |
| *fliQ* | flagellar biosynthetic FliQ protein | -2.05 | 0.00 |
| *fliP* | flagellar biosynthetic protein FliP precursor protein | -2.09 | 0.03 |
| *fliO ^#^* | flagellar biogenesis FliO protein | -2.73 | 0.00 |
| *fliK ^#^* | flagellar hook-length control protein | -2.60 | 0.00 |
| *fliJ ^#^* | flagellar protein | -2.48 | 0.00 |
| *fliI ^#^* | flagellum-specific ATP synthase protein | -3.46 | 0.00 |
| *fliH** | flagellar assembly FliH protein | -1.75 | 0.00 |
| *fliG ^#^* | flagellar motor switch FliG protein | -2.35 | 0.01 |
| Hsero_2059 ^#^ | hypothetical protein | -2.59 | 0.00 |
| Hsero_2072 | TPR repeat containing protein | -2.23 | 0.00 |
| *phoH* | Phosphate starvation-inducible ATPase protein | -2.06 | 0.00 |
| *bcp* | bacterioferritin comigratory oxidoreductase protein | -2.06 | 0.00 |
| Hsero_2084* | xylanase/chitin deacetylase protein | -1.21 | 0.31 |
| Hsero_2111* | signal transduction histidine kinase protein | -1.33 | 0.04 |
| *menG* | S-adenosylmethionine:2-demethylmenaquinone methyltransferase protein | -2.12 | 0.00 |
| *aceA ^#^* | isocitrate lyase protein | 3.22 | 0.01 |
| *modC1** | ABC-type sulfate/molybdate transport systems. ATPase component protein | -1.47 | 0.33 |
| *modB1** | ABC-type molybdate transport system. permease component protein | 1.05 | 0.90 |
| *modA1* | ABC-type molybdate transport system. periplasmic component protein | -2.35 | 0.00 |
| Hsero_2136* | signal transduction containing EAL and modified HD-GYP domains protein | -1.31 | 0.12 |
| Hsero_2137 | signal transduction containing PAS/GGDEF/EAL domains protein | -2.07 | 0.00 |
| *sodC ^#^* | sodC Cu/Zn superoxide dismutase protein | -2.80 | 0.00 |
| Hsero_2155 | hypothetical protein | -2.64 | 0.02 |
| *cphA2* | cyanophycin synthetase protein | -2.26 | 0.00 |
| *cphA1** | cyanophycin synthetase protein | -1.63 | 0.06 |
| *smpB** | tmRNA-binding protein | 1.11 | 0.83 |
| Hsero_2203* | oligoketide cyclase/lipid transport protein | -1.17 | 0.07 |
| Hsero_2204 | conserved hypothetical protein | -2.59 | 0.02 |
| *guaB** | inosine-5'-monophosphate dehydrogenase protein | -1.55 | 0.05 |
| Hsero_2222 | plasmid stabilization protein | 2.57 | 0.02 |
| Hsero_2246 | peptide methionine sulfoxide reductase protein | -2.07 | 0.00 |
| Hsero_2247* | conserved hypothetical protein | -1.57 | 0.20 |
| Hsero_2252 ^#^ | permease major facilitator superfamily MFS_1 protein | -2.28 | 0.00 |
| Hsero_2292 ^#^ | hypothetical protein | -3.11 | 0.00 |
| Hsero_2321 ^#^ | conserved hypothetical protein | -2.74 | 0.00 |
| *csdB** | selenocysteine lyase protein | 3.28 | 0.09 |
| Hsero_2333 ^#^ | major membrane protein I (35 kDa antigen) protein | 5.02 | 0.03 |
| Hsero_2334 | conserved hypothetical protein | # | 0.10 |
| *cysE ** | serine O-acetyltransferase protein | 1.72 | 0.58 |
| Hsero_2359 ^#^ | bacteriophage replication protein | 2.57 | 0.01 |
| Hsero_2360 ^#^ | hypothetical protein | 2.17 | 0.00 |
| Hsero_2361* | hypothetical protein | 1.77 | 0.33 |
| Hsero_2362* | hypothetical protein | 1.39 | 0.64 |
| Hsero_2363 | hypothetical protein | 2.40 | 0.01 |
| Hsero_2364** | hypothetical protein | 2.84 | 0.31 |
| Hsero_2365 ^#^ | conserved hypothetical protein | 2.38 | 0.01 |
| Hsero_2367 ^#^ | hypothetical protein | 3.37 | 0.00 |
| Hsero_2368**^#^ | hypothetical protein | 2.63 | 0.18 |
| Hsero_2369* | soluble lytic murein transglycosylase protein | 1.43 | 0.44 |
| Hsero_2370* | hypothetical protein | 1.02 | 0.92 |
| Hsero_2371 ^#^ | hypothetical protein | 3.29 | 0.00 |
| Hsero_2372 ^#^ | conserved hypothetical protein | -2.56 | 0.01 |
| Hsero_2373* | conserved hypothetical protein | -1.93 | 0.05 |
| Hsero_2390* | flavodoxin protein | 1.51 | 0.43 |
| Hsero_2391 ^#^ | conserved hypothetical protein | 3.06 | 0.00 |
| Hsero_2393* | periplasmic or secreted lipoprotein | 1.92 | 0.00 |
| Hsero_2394 | hypothetical protein | 3.69 | 0.00 |
| Hsero_2408 | hypothetical protein | 3.47 | 0.04 |
| Hsero_2409* | transcription regulator. LysR family protein | 1.28 | 0.62 |
| *tam* | trans-aconitate methyltransferase protein | 2.27 | 0.00 |
| Hsero_2435 ^#^ | transcription response regulator protein | -3.09 | 0.00 |
| *baeS ** | signal transduction histidine kinase protein | -1.16 | 0.65 |
| Hsero_2465 | permease of the major facilitator superfamily protein | -2.54 | 0.00 |
| Hsero_2469 ^#^ | conserved hypothetical protein | -4.41 | 0.00 |
| Hsero_2496* | membrane protein | 1.46 | 0.72 |
| *pgi* | glucose-6-phosphate isomerase protein | 3.82 | 0.01 |
| Hsero_2518**^#^ | conserved hypothetical protein | 2.13 | 0.15 |
| Hsero_2519 ^#^ | amino acid transporter permease protein | 2.41 | 0.00 |
| *tauC *^#^* | ABC-type nitrate/sulfonate/bicarbonate transport system. permease component protein | -1.79 | 0.01 |
| *tauB* | ABC-type nitrate/sulfonate/bicarbonate transport system. ATPase component protein | -2.09 | 0.00 |
| *fnr ** | Crp/Fnr family transcription regulator protein | -1.37 | 0.09 |
| Hsero_2539* | type 2 phosphatidic acid phosphatase family protein | -1.57 | 0.06 |
| Hsero_2540* | sphingosine kinase/eukaryotic diacylglycerol kinase protein | -1.61 | 0.01 |
| *cpdA* | 3'.5'-cyclic-nucleotide phosphodiesterase protein | -2.08 | 0.04 |
| *paaK ^#^* | phenylacetyl-CoA-ligase protein | -6.48 | 0.00 |
| Hsero_2562 ^#^ | ABC-type amino acid transport system. ATPase component protein | -7.49 | 0.00 |
| Hsero_2563 ^#^ | ABC-type amino acid transport system. periplasmic component protein | -9.36 | 0.00 |
| Hsero_2564 ^#^ | ABC-type amino acid transport system. permease component protein | -7.37 | 0.00 |
| Hsero_2565 ^#^ | ABC-type amino acid transport system. permease component protein | -10.92 | 0.00 |
| Hsero_2566 ^#^ | ABC-type amino acid transport system protein. ATPase component protein | -6.30 | 0.00 |
| *fadD ^#^* | long-chain acyl-CoA synthetase protein | -6.03 | 0.00 |
| *papD** | PilP assembly chaperone transmembrane protein | 1.00 | 0.27 |
| *papC* | P pilus assembly protein. porin PapC protein | -2.03 | 0.04 |
| Hsero_2576 | spore coat U domain protein | -2.09 | 0.00 |
| *lldD* | L-lactate dehydrogenase protein | -2.82 | 0.00 |
| Hsero_2616 | intracellular protease/amidase protein | -2.01 | 0.00 |
| *dctA ^#^* | C4-dicarboxylate transport transmembrane protein | -2.89 | 0.01 |
| *tsr* | methyl-accepting chemotaxis II protein | -2.06 | 0.00 |
| *phaC ^#^* | phaC poly(3-hydroxyalkanoate) synthetase protein | -2.77 | 0.00 |
| *cobQ* | cobyric acid synthase protein | -2.60 | 0.00 |
| *cobC* | cobalamin biosynthesis protein | -2.21 | 0.01 |
| *cobD ^#^* | cobalamin biosynthesis (CobD/CbiB) protein | -11.43 | 0.00 |
| *cobP** | bifunctional enzyme: cobinamide kinase/cobinamide phosphate guanylyltransferase protein | -1.87 | 0.35 |
| *nfnB* | oxygen-insensitive nitroreductase protein | -3.50 | 0.00 |
| *cobB ^#^* | cobyrinic acid A.c-diamide synthase protein | -3.80 | 0.00 |
| *cobA** | cob(I)alamin adenosyltransferase protein | -1.77 | 0.00 |
| Hsero_2648 | G3E family GTPase protein | -2.48 | 0.00 |
| *cbiJH** | bifunctional: precorrin-3 methyltransferase and precorrin-6x reductase oxidoreductase protein 3023458:3025017 reverse | -1.82 | 0.00 |
| *cbiG ^#^* | bifunctional precorrin-3B C17-methyltransferase/precorrin isomerase protein | -2.73 | 0.01 |
| *cbiF* | precorrin-4 C11-methyltransferase protein | -2.22 | 0.00 |
| *cbiL ^#^* | precorrin-2 C20-methyltransferase protein | -3.08 | 0.01 |
| *cbiD* | cobalamin biosynthesis CbiD transmembrane protein | -2.45 | 0.00 |
| *cobH ^#^* | precorrin-8X methylmutase protein | -2.76 | 0.00 |
| *cobL* | precorrin-6y methylase methyltransferase protein | -2.09 | 0.00 |
| *chlI ^#^* | Mg-chelatase subunit ChlI protein | -2.69 | 0.01 |
| *cobN* | cobalamin biosynthesis protein | -2.05 | 0.01 |
| *galU ^#^* | UTP-glucose-1-phosphate uridylyltransferase protein | 2.39 | 0.03 |
| *serA* | D-3-phosphoglycerate dehydrogenase protein | -2.14 | 0.00 |
| *fabD ^#^* | malonyl CoA-[acyl-carrier-protein] transacylase protein | -3.67 | 0.00 |
| *mdcB* | triphosphoribosyl-dephospho-CoA synthetase protein | -2.11 | 0.02 |
| *mdcG ^#^* | phosphoribosyl-dephospho-CoA transferase protein | -3.40 | 0.00 |
| *mdcC ^#^* | malonate decarboxylase gamma-subunit protein | -2.59 | 0.00 |
| *accD* | acetyl-CoA carboxylase beta subunit protein | -2.23 | 0.00 |
| *mdcA* | malonate decarboxylase alpha subunit protein | -2.13 | 0.00 |
| *dctM* | TRAP-type C4-dicarboxylate transport system. large permease component protein | -2.24 | 0.00 |
| *dctQ ^#^* | TRAP-type C4-dicarboxylate transport system. small permease component protein | -2.48 | 0.00 |
| *dctP* | TRAP-type C4-dicarboxylate transport system. periplasmic component protein | -2.24 | 0.00 |
| Hsero_2695 | transmembrane transcription regulator (anti-sigma factor) protein | -2.45 | 0.00 |
| *rpoE* * | RNA polymerase sigma-E factor (sigma-24) | -1.88 | 0.12 |
| Hsero_2707 | oxidoreductase protein | -2.03 | 0.01 |
| Hsero_2708 ^#^ | D-galactose-1-dehydrogenase protein | -3.04 | 0.00 |
| Hsero_2768* | nicotinamide mononucleotide transporter protein | -1.97 | 0.02 |
| *ftsB ^#^* | cell division. septum formation initiator. FtsB protein | -2.45 | 0.05 |
| *eno*** | enolase (2-Phosphoglycerate dehydratase) protein | -1.40 | 0.09 |
| Hsero_2800 | LysR family transcription regulator protein | -4.20 | 0.00 |
| *metC* | cystathionine beta-lyase (cysteine lyase) protein 3405203:3406381 reverse | -2.16 | 0.00 |
| *phbB* | acetoacetyl-CoA reductase protein | -2.11 | 0.00 |
| Hsero_3031 ^#^ | beta-lactamase class C protein | -2.40 | 0.00 |
| *tsr* * | methyl-accepting chemotaxis transducer transmembrane protein | -1.31 | 0.01 |
| *mmsB ^#^* | 3-hydroxyisobutyrate dehydrogenase protein | 2.93 | 0.00 |
| *mmsA^#^* | methylmalonate-semialdehyde dehydrogenase protein | 2.97 | 0.01 |
| Hsero_3068* | ABC-type branched-chain amino acid transport system.periplasmic component protein | 1.48 | 0.39 |
| Hsero_3075 | FAD-dependent monooxygenase protein | -1.96 | 0.01 |
| *caiD** | enoyl-CoA hydratase/carnithine racemase protein | -1.37 | 0.35 |
| *trnE* | tRNA-Glu | 5.24 | 0.00 |
| *cheZ ** | chemotaxis phosphatase protein | -1.28 | 0.02 |
| *cheA* | chemotaxis CheA protein | -2.00 | 0.00 |
| *prpC ** | methylcitrate synthase (Citrate synthase 2) protein | -1.22 | 0.59 |
| *prpB* | carboxyphosphonoenolpyruvate phosphonomutase protein | -2.03 | 0.00 |
| *ntrC ^#^* | nitrogen assimilation transcription activator protein | 4.14 | 0.00 |
| *ntrB**^#^* | nitrogen regulation (sensor histidine kinase) transcription regulator protein | 2.23 | 0.09 |
| *glnA* | glutamine synthetase protein | 2.03 | 0.04 |
| Hsero_3195 ^#^ | fatty acid desaturase protein | -2.66 | 0.00 |
| Hsero_3196 ^#^ | hypothetical protein | -2.40 | 0.00 |
| *fnr* | Crp/Fnr family transcription regulator protein | -2.26 | 0.00 |
| Hsero_3198 ^#^ | transmembrane protein | -3.21 | 0.01 |
| Hsero_3199** | conserved hypothetical protein | -1.98 | 0.16 |
| *fixG** | iron-sulfur 4Fe-4S ferredoxin transmembrane protein | -1.20 | 0.12 |
| *fixP** | cbb3-type cytochrome c oxidase. subunit III transmembrane protein | -1.37 | 0.13 |
| *fixO** | cbb3-type cytochrome c oxidase. subunit II transmembrane protein | -1.16 | 0.46 |
| *fixN** | cbb3-type cytochrome c oxidase. subunit I transmembrane protein | -1.50 | 0.05 |
| *fixS* | nitrogen fixation protein | -2.82 | 0.00 |
| *fixI** | cation transport P-type ATPase protein | -1.54 | 0.13 |
| Hsero_3206 | conserved hypothetical protein | 1.06 | 0.81 |
| *hemN ** | oxygen-independent coproporphyrinogen III oxidase protein | -1.48 | 0.04 |
| *Hsero_3214* | sensory transduction regulatory protein | -2.12 | 0.01 |
| *chaC** | cation transport regulator protein | -1.87 | 0.04 |
| Hsero_3227* | hypothetical protein | 1.17 | 0.80 |
| Hsero_3228 ^#^ | hypothetical protein | 3.16 | 0.03 |
| Hsero_3229** | hypothetical protein | 8.17 | 0.15 |
| Hsero_3230** | hypothetical protein | 7.60 | 0.09 |
| Hsero_3234 ^#^ | methyl-accepting chemotaxis transducer transmembrane protein | -2.62 | 0.00 |
| *xdhA** | xanthine dehydrogenase (subunit A) protein | -1.30 | 0.05 |
| *xdhB* | xanthine dehydrogenase (subunit B) protein | -2.11 | 0.00 |
| *xdhC* | xanthine/CO dehydrogenase maturation factor | -2.13 | 0.01 |
| Hsero_3303 ^#^ | CBS domain containing protein | -2.48 | 0.00 |
| *phaB ^#^* | 3-ketoacyl-CoA reductase protein | -2.81 | 0.00 |
| Hsero_3329 ^#^ | GntR family transcription regulator protein | -3.07 | 0.00 |
| Hsero_3330 ^#^ | malate/L-lactate dehydrogenase protein | -4.02 | 0.00 |
| Hsero_3331 ^#^ | transmembrane protein | -3.46 | 0.00 |
| Hsero_3332 ^#^ | conserved hypothetical protein | -3.22 | 0.00 |
| Hsero_3333 ^#^ | conserved hypothetical protein | -2.81 | 0.03 |
| Hsero_3334* | hypothetical protein | -1.50 | 0.34 |
| *ilvD* | dihydroxyacid dehydratase/phosphogluconate dehydratase protein | -2.18 | 0.00 |
| *serA* | D-3-phosphoglycerate dehydrogenase protein | -2.12 | 0.00 |
| Hsero_3337 | ABC-type sugar transport systems. ATPase component protein | -2.06 | 0.00 |
| Hsero_3338 ^#^ | ABC-type sugar transport system. periplasmic component protein | -4.14 | 0.00 |
| Hsero_3339 ^#^ | ABC-type sugar transport system. permease component protein | -2.84 | 0.00 |
| Hsero_3340 ^#^ | ABC-type sugar transport system. permease component protein | -4.11 | 0.00 |
| Hsero_3345 | transmembrane protein | -2.13 | 0.01 |
| Hsero_3346 | Zn-dependent protease with chaperone function transmembrane protein | -2.11 | 0.01 |
| *penP ^#^* | beta-lactamase class A protein | -10.42 | 0.00 |
| *cinA1* | competence- and mitomycin-induced protein | -2.02 | 0.04 |
| *mgtA* | Mg(2+) transport ATPase. P-type 1 protein | 4.77 | 0.00 |
| Hsero_3382* | periplasmic sensor signal transduction histidine kinase protein | -1.54 | 0.02 |
| Hsero_3383 | two component response regulator protein | -2.16 | 0.01 |
| Hsero_3420 ^#^ | ABC-type uncharacterized transport system. duplicated ATPase component protein | -2.42 | 0.01 |
| Hsero_3421 ^#^ | ABC-type uncharacterized transport system. permease component protein | -2.76 | 0.00 |
| Hsero_3422* | ABC-type uncharacterized transport system. permease component protein | -1.76 | 0.07 |
| Hsero_3423* | ABC-type oligopeptide transport system. periplasmic component protein | -1.95 | 0.00 |
| *chrA ^#^* | chromate transport protein | 3.01 | 0.03 |
| *chrA*^#^* | chromate transport protein | 1.92 | 0.06 |
| Hsero_3478 | hypothetical protein | 2.47 | 0.01 |
| Hsero_3492 ^#^ | hypothetical protein | -3.60 | 0.00 |
| *glnH ** | ABC-type glutamine transport system. periplasmic component protein | 1.94 | 0.22 |
| *glnP* | ABC-type glutamine transport system. permease component protein | 2.84 | 0.02 |
| *glnQ ** | ABC-type glutamine transport system. ATPase component protein | -1.07 | 0.52 |
| Hsero_3592* | acetyltransferase protein | 1.63 | 0.33 |
| Hsero_3593**^#^ | oxacillin hydrolase (class-D beta-lactamase) signal peptide protein | 2.25 | 0.09 |
| Hsero_3594 | cytosolic protein | 5.52 | 0.03 |
| Hsero_3595* | carboxylesterase protein | 1.45 | 0.31 |
| Hsero_3596* | hypothetical protein | 1.38 | 0.31 |
| Hsero_3626 | extracellular polysaccharide synthase protein | -2.15 | 0.02 |
| Hsero_3627 | hypothetical protein | -2.44 | 0.00 |
| Hsero_3632 ^#^ | peroxidase-related protein | -4.03 | 0.00 |
| *ribD** | bifunctional enzyme: diaminohydroxyphosphoribosyl aminopyrimidine deaminase/5-amino-6-(5-phosphoribosylamino) uracil reductase protein | -1.87 | 0.11 |
| *ribE* | riboflavin synthase (alpha chain) protein | -2.30 | 0.00 |
| *caiB** | acyl-CoA transferases/carnitine dehydratase protein | -1.09 | 0.14 |
| *recR** | rocombinational DNA repair protein | -1.69 | 0.20 |
| Hsero_3679* | conserved hypothetical protein | -1.70 | 0.34 |
| Hsero_3680* | conserved hypothetical protein | -1.22 | 0.01 |
| *dnaX** | DNA polymerase III (subunits tau and gamma) protein | -1.52 | 0.04 |
| Hsero_3682 | GGDEF family protein | -2.18 | 0.00 |
| *fabG ^#^* | 3-oxoacyl-(acyl-carrier-protein) reductase protein | -2.51 | 0.00 |
| *fabF ** | 3-oxoacyl-[acyl-carrier-protein] synthase II protein | -1.48 | 0.45 |
| Hsero_3717 | excinuclease ATPase subunit protein | -2.52 | 0.00 |
| Hsero_3718* | phosphopantetheinyl transferase protein | -1.30 | 0.76 |
| Hsero_3719 ^#^ | hypothetical protein | -4.11 | 0.00 |
| Hsero_3735 | two component hybrid sensor histidine kinase/response regulator protein | -2.05 | 0.00 |
| Hsero_3736 | two component response regulator protein | -2.01 | 0.00 |
| Hsero_3754 | hypothetical protein | -2.26 | 0.01 |
| *cheD3 ^#^* | methyl-accepting chemotaxis I protein | -2.45 | 0.00 |
| *ilvC* | ketol-acid reductoisomerase oxidoreductase protein | -2.52 | 0.01 |
| Hsero_3807* | Hsero_3807 sigma54-dependent transcription regulator protein 4359701:4361014 reverse | -1.94 | 0.04 |
| fabG | short-chain alcohol dehydrogenase protein | -3.14 | 0.00 |
| *fabG** | 3-oxoacyl-(acyl-carrier-protein) reductase protein | -1.52 | 0.04 |
| Hsero_3817* | TRAP-type C4-dicarboxylate transport system. dctQ-M fusion permease. large permease component protein | -1.35 | 0.00 |
| Hsero_3818* | TRAP-type C4-dicarboxylate transport system. periplasmic component protein | -1.46 | 0.02 |
| fadD* | long-chain-fatty-acid-CoA ligase protein | 1.00 | 0.76 |
| Hsero_3822* | conserved hypothetical protein | -1.15 | 0.67 |
| Hsero_3823 | conserved hypothetical protein | -2.22 | 0.02 |
| *glcF ^#^* | glycolate oxidase. iron-sulfur subunit oxidoreductase protein | -4.63 | 0.00 |
| *glcE ^#^* | glycolate oxidase iron-sulfur subunit oxidoreductase protein | -3.72 | 0.00 |
| *glcD ^#^* | glycolate oxidase (FAD-linked subunit) oxidoreductase protein | -3.50 | 0.00 |
| *gdhA ^#^* | glutamate dehydrogenase (NAD(P)+) protein | -3.59 | 0.00 |
| *fadD* | long-chain-fatty-acid-CoA ligase protein | -2.08 | 0.01 |
| Hsero_3877 | fumarylacetoacetate hydrolase family protein | -2.04 | 0.01 |
| *fabG* | short-chain alcohol dehydrogenase protein | -1.77 | 0.11 |
| Hsero_3899* | permease of the major facilitator superfamily protein | -1.48 | 0.13 |
| *ubiB** | ubiquinone biosynthesis transmembrane protein | -1.73 | 0.04 |
| Hsero_3901* | conserved hypothetical protein | -1.15 | 0.70 |
| Hsero_3902* | transmembrane protein | -1.19 | 0.53 |
| *ubiE** | ubiquinone/menaquinone biosynthesis; 2-octaprenyl-6-methoxy-1.4-benzoquinonemethyltransferase protein | -1.17 | 0.33 |
| Hsero_3904* | conserved hypothetical protein | -1.05 | 0.79 |
| *hit* | diadenosine tetraphosphate hydrolase protein | -2.26 | 0.05 |
| Hsero_3906* | oxidoreductase protein | -1.45 | 0.12 |
| *ptsN** | nitrogen regulatory IIA (Enzyme IIA-NTR) (Phosphotransferase enzyme II. A component) transcription regulator protein | -1.20 | 0.37 |
| *ptsK* | Hpr kinase and phosphatase protein | -2.22 | 0.00 |
| Hsero_3913* | P-loop-containing kinase protein | -1.51 | 0.01 |
| Hsero_3977 ^#^ | short-chain alcohol dehydrogenase protein | -2.80 | 0.00 |
| Hsero_3980 ^#^ | response regulator transcription regulator protein 4566029:4566595 reverse | -3.31 | 0.00 |
| pcaR | transcription regulator protein | 2.71 | 0.00 |
| *livF ^#^* | ABC-type branched-chain amino acid transport system. ATPase component protein | -2.27 | 0.00 |
| *livG ^#^* | ABC-type branched-chain amino acid transport system. ATPase component protein | -2.03 | 0.00 |
| *livM** | ABC-type branched-chain amino acid transport system. permease component protein | -1.40 | 0.08 |
| *livH** | ABC-type branched-chain amino acid transport system. permease component protein | -1.05 | 0.84 |
| *pcaF ^#^* | beta-ketoadipyl-CoA thiolase protein | 7.20 | 0.00 |
| *pcaJ ^#^* | 3-oxoadipate CoA-transferase subunit B protein | 6.03 | 0.01 |
| *pcaI ^#^* | 3-oxoadipate CoA-transferase subunit A protein | 7.09 | 0.01 |
| *pcaR* | IclR family transcription regulator protein | -2.14 | 0.00 |
| *ompC ^#^* | porin transmembrane protein | 2.43 | 0.04 |
| Hsero_4005 | ABC-type branched-chain amino acid transport system. periplasmic component protein | 2.41 | 0.00 |
| Hsero_4011* | ABC-type branched-chain amino acid transport system. periplasmic component protein | -1.59 | 0.00 |
| Hsero_4012* | ABC-type branched-chain amino acid transport system. permease component protein | -1.84 | 0.12 |
| Hsero_4013 | ABC-type branched-chain amino acid transport system. permease component protein | -2.19 | 0.04 |
| Hsero_4014* | ABC-type branched-chain amino acid transport system. ATPase component protein | -1.55 | 0.09 |
| Hsero_4015 ^#^ | conserved hypothetical protein | -4.31 | 0.00 |
| *aidB* | acyl-CoA dehydrogenase protein | -2.71 | 0.00 |
| Hsero_4017* | MarR family transcription regulator protein | -1.08 | 0.78 |
| Hsero_4020 | gluconolactonase protein | -2.12 | 0.01 |
| Hsero_4021 | ABC-type sugar transport system. permease component protein | -3.52 | 0.00 |
| Hsero_4022 | ABC-type sugar transport system. permease component protein | -2.44 | 0.01 |
| Hsero_4023* | ABC-type sugar transport system. ATPase component protein | -1.23 | 0.47 |
| Hsero_4024* | ABC-type sugar transport system. periplasmic component protein | -1.30 | 0.15 |
| Hsero_4027* | transmembrane protein | 1.75 | 0.02 |
| Hsero_4028 | conserved hypothetical protein | 2.83 | 0.00 |
| Hsero_4029* | lipoprotein | 1.09 | 0.78 |
| *petC** | cytochrome c1 precursor transmembrane protein | -1.22 | 0.00 |
| *petB* | cytochrome b subunit transmembrane protein | -2.04 | 0.00 |
| *petA** | transmembrane ubiquinol-cytochrome C reductase (Iron-sulfur subunit) oxidoreductase protein | -1.02 | 0.66 |
| *tatC* | Sec-independent protein translocase protein | -2.98 | 0.00 |
| *tatB** | Sec-independent protein translocase protein | -1.41 | 0.39 |
| *coxC* | cytochrome c oxidase subunit III transmembrane protein | -2.24 | 0.00 |
| Hsero_4158 | transmembrane protein | -2.22 | 0.00 |
| *coxG** | cytochrome C oxidase assembly transmembrane protein | -1.38 | 0.00 |
| Hsero_4278 ^#^ | indolepyruvate ferredoxin oxidoreductase. alpha and beta subunits protein | -2.85 | 0.00 |
| Hsero_4300 | conserved hypothetical protein | -2.03 | 0.03 |
| *pheC* | arogenate dehydratase protein | -2.02 | 0.00 |
| *rfbG* | CDP-glucose-4.6-dehydratase protein | -2.03 | 0.00 |
| *galE** | UDP-glucose 4-epimerase protein | -1.98 | 0.20 |
| *rfbB* | dTDP-glucose 4.6-dehydratase protein | -2.37 | 0.02 |
| *rfbC** | dTDP-6-deoxy-D-glucose-3.5 epimerase protein | -1.75 | 0.24 |
| Hsero_4412* | hypothetical protein | -1.77 | 0.00 |
| Hsero_4413 | galactokinase/mevalonate kinase protein | -2.04 | 0.01 |
| *wcaG* | GDP-4-keto-6-deoxy-D-mannose-3. 5-epimerase-4-reductase protein | -3.24 | 0.00 |
| Hsero_4415 ^#^ | CDP-4-keto-6-deoxy-D-glucose-3-dehydrase protein | -2.45 | 0.00 |
| Hsero_4416 | phospho-sugar nucleotidyltransferase protein | -2.18 | 0.00 |
| Hsero_4417 | sedoheptulose 7-phosphate isomerase protein | -2.09 | 0.00 |
| Hsero_4418 ^#^ | histidinol phosphatase protein | -4.16 | 0.00 |
| Hsero_4419 ^#^ | hypothetical protein | -3.65 | 0.00 |
| Hsero_4420* | SAM-dependent methyltransferase protein | -1.93 | 0.02 |
| Hsero_4421 ^#^ | hypothetical protein | -2.39 | 0.00 |
| *wcaA* | glycosyltransferase protein | -2.00 | 0.00 |
| *xylF ^#^* | ABC-type D-xylose transport system. periplasmic component protein | -3.05 | 0.00 |
| *xylG ^#^* | ABC-type D-xylose transport system. ATPase component protein | -2.87 | 0.00 |
| *xylH ^#^* | ABC-type D-xylose transport system. permease component protein | -4.12 | 0.00 |
| Hsero_4525* | AraC family transcription regulator protein | 1.39 | 0.49 |
| Hsero_4526* | transcription regulator protein | 1.81 | 0.23 |
| Hsero_4527 | conserved hypothetical protein | 2.42 | 0.00 |
| Hsero_4543 ^#^ | methyl-accepting chemotaxis transmembrane protein | -3.23 | 0.00 |
| Hsero_4615 ^#^ | methyl-accepting chemotaxis protein I. serine sensor receptor protein | -2.44 | 0.00 |
| Hsero_4622** | cytochrome c2 protein | -4.14 | 0.08 |
| Hsero_4623 | conserved hypothetical protein | -2.11 | 0.02 |
| *atoA* | acyl CoA:acetate/3-ketoacid CoA transferase. beta subunit protein | -2.03 | 0.00 |
| *atoD* | acyl CoA:acetate/3-ketoacid CoA transferase. alpha subunit protein | -2.15 | 0.00 |
| *livK* | ABC-type branched-chain amino acid transport system. periplasmic component protein | -2.12 | 0.00 |
| *livH* | ABC-type branched-chain amino acid transport system. permease component protein | -2.15 | 0.01 |
| *livM ** | ABC-type branched-chain amino acid transport system. permease component protein | -1.67 | 0.03 |
| *livG ^#^* | ABC-type branched-chain amino acid transport system. ATPase component protein | -3.91 | 0.00 |
| *livF* | ABC-type branched-chain amino acid transport system. ATPase component protein | -2.06 | 0.03 |
| *hisC* | histidinol-phosphate aminotransferase 1 protein | -2.13 | 0.00 |
| Hsero_4702 ^#^ | conserved hypothetical protein | -3.55 | 0.00 |
| Hsero_4704 ^#^ | acyl-CoA synthetase (AMP-forming)/AMP-acid ligase II protein | -4.25 | 0.01 |
| urtA ^#^ | ABC-type urea transport system. periplasmic component protein | 3.44 | 0.01 |
| urtB ^#^ | ABC-type urea transport system. permease component protein | 2.52 | 0.03 |
| urtC** | urea transport system. permease component protein | 2.10 | 0.17 |
| urtD ^#^ | urea transport system. ATPase component protein | 3.52 | 0.00 |
| urtE | ABC-type urea transport system. ATPase component protein | 4.14 | 0.00 |
| Hsero_4759 ^#^ | conserved hypothetical protein | 2.33 | 0.04 |
| Hsero_4778 | branched-chain amino acid aminotransferase/4-amino-4-deoxychorismate lyase protein | -2.32 | 0.00 |
| Hsero_4792 | ABC-type amino acid transport system. permease component protein | -2.23 | 0.00 |
| Hsero_4793^#^ | ABC-type branched-chain amino acid transport system. permease component protein | -2.59 | 0.00 |
| Hsero_4794 | ABC-type amino acid transport system. periplasmic component protein | -2.27 | 0.00 |
| Hsero_4795 ^#^ | ABC-type amino acid transport system. ATPase component protein | -2.43 | 0.00 |
| Hsero_4796 | ABC-type amino acid transport system. ATPase component protein | -2.32 | 0.00 |
| *tauA* | ABC-type nitrate/sulfonate/bicarbonate transport system. periplasmic component protein | -2.04 | 0.00 |

Three hundred and eigth one genes were regulated by naringenin (Fold change ≥2 or ≤-2 and p<0.05; Baggerley´s test). When the gene regulated was founded in an operon, all genes of the operon are shaded in gray. *Fold change ≤ 2; ** p value > 0.05; ^#^ Fold change ≥2 or ≤-2 and p value ≤ 0.05 by the DESeq.
