# Supplementary figures and images for: Genome wide transcriptional profiling of Herbaspirillum seropedicae SmR1 grown in the presence of naringenin
Source: Front Microbiol. 2015 May 21;6:491. doi: 10.3389/fmicb.2015.00491 (PMC4440368; doi:10.3389/fmicb.2015.00491)

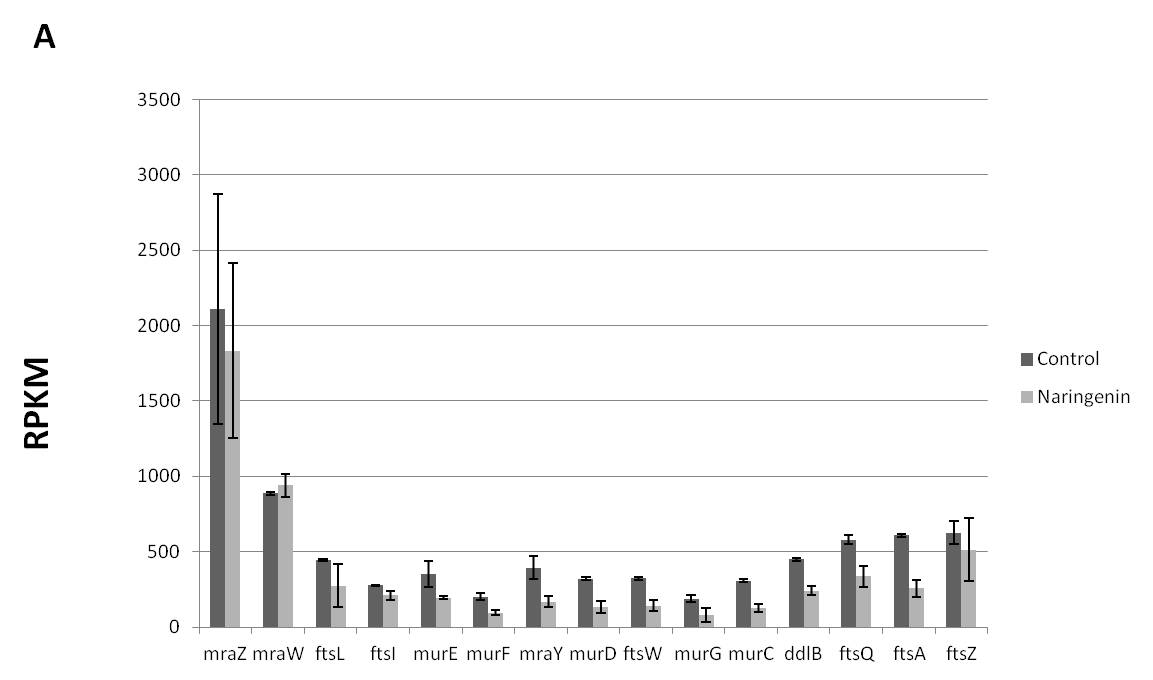

Supplement: Supplementary file 2 [file Image1.JPEG]

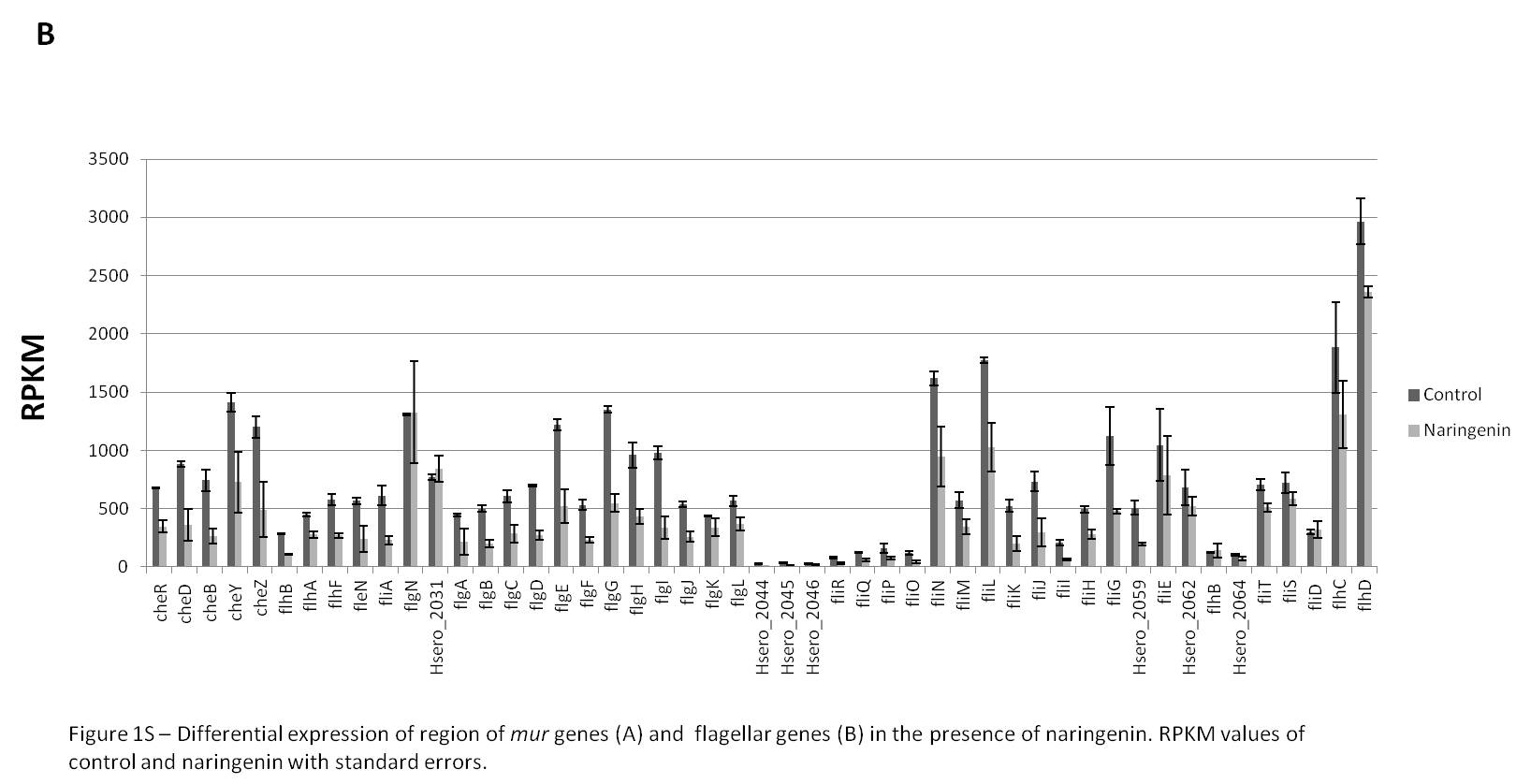

Supplement: Supplementary file 3 [file Image2.JPEG]
